# Objective Emotion Assessment Using a Triple Attention Network for an EEG-Based Brain–Computer Interface

**DOI:** 10.3390/brainsci15111167

**Published:** 2025-10-29

**Authors:** Lihua Zhang, Xin Zhang, Xiu Zhang, Changyi Yu, Xuguang Liu

**Affiliations:** 1 Tianjin Key Laboratory of Wireless Mobile Communications and Power Transmission, Tianjin Normal University, Tianjin 300387, Chinaecezhang@tjnu.edu.cn (X.Z.);; 2 College of Artificial Intelligence, Tianjin Normal University, Tianjin 300387, China

**Keywords:** emotion assessment, electroencephalography, brain–computer interface, deep learning, attention mechanism

## Abstract

**Background:** The assessment of emotion recognition holds growing significance in research on the brain–computer interface and human–computer interaction. Among diverse physiological signals, electroencephalography (EEG) occupies a pivotal position in affective computing due to its exceptional temporal resolution and non-invasive acquisition. However, EEG signals are inherently complex, characterized by substantial noise contamination and high variability, posing considerable challenges to accurate assessment. **Methods:** To tackle these challenges, we propose a Triple Attention Network (TANet), a triple-attention EEG emotion recognition framework that integrates Conformer, Convolutional Block Attention Module (CBAM), and Mutual Cross-Modal Attention (MCA). The Conformer component captures temporal feature dependencies, CBAM refines spatial channel representations, and MCA performs cross-modal fusion of differential entropy and power spectral density features. **Results:** We evaluated TANet on two benchmark EEG emotion datasets, DEAP and SEED. On SEED, using a subject-specific cross-validation protocol, the model reached an average accuracy of 98.51 ± 1.40%. On DEAP, we deliberately adopted a segment-level splitting paradigm—in line with influential state-of-the-art methods—to ensure a direct and fair comparison of model architecture under an identical evaluation protocol. This approach, designed specifically to assess fine-grained within-trial pattern discrimination rather than cross-subject generalization, yielded accuracies of 99.69 ± 0.15% and 99.67 ± 0.13% for the valence and arousal dimensions, respectively. Compared with existing benchmark approaches under similar evaluation protocols, TANet delivers substantially better results, underscoring the strong complementary effects of its attention mechanisms in improving EEG-based emotion recognition performance. **Conclusions:** This work provides both theoretical insights into multi-dimensional attention for physiological signal processing and practical guidance for developing high-performance, robust EEG emotion assessment systems.

## 1. Introduction

Over the past decade, emotion assessment has become pivotal enabling technology in domains such as the brain–computer interface (BCI) and human–computer interaction (HCI), intelligent healthcare, personalized recommendations, and mental health monitoring [1]. In contrast to visible expressions like facial gestures or vocal intonation, EEG records neural activity patterns directly, allowing assessment of emotional states with strengths such as fine-grained temporal resolution, non-invasive measurement, and portability in deployment devices [2]. Owing to these benefits, EEG-based emotion recognition has attracted considerable interest in affective computing and BCI research [3,4]. Although EEG signals provide rich emotional information, their high-dimensional, non-stationary, multi-channel nature, and significant inter-individual variability pose significant challenges for feature extraction and emotion modeling [5]. Early research primarily relied on handcrafted feature descriptors combined with shallow classification algorithms. Conventional methods typically depended on hand-crafted features, including event-related potentials [6] in the time domain, power spectral density (PSD) [7] in the frequency domain, and nonlinear measures like differential entropy (DE) features [8], paired with classifiers such as Support Vector Machines (SVM) [9], K-Nearest Neighbors [10], and random forests (RF) [11]. For instance, Koelstra et al. achieved preliminary success using PSD features and SVM on the DEAP dataset [12], while Zheng et al. demonstrated the potential of DE features with an RF classifier on the SEED dataset [13]. While these methods established the feasibility of the task, they exhibited limited performance when handling high-complexity signals [14] and struggled to capture the complex, non-stationary dynamics inherent in EEG.

In recent years, deep learning has emerged as the dominant paradigm to overcome the limitations of traditional methods in processing complex and heterogeneous datasets, making it highly relevant to EEG-based affective computing. Convolutional neural networks (CNNs), such as the compact EEGNet architecture developed by Lawhern et al. [15], have been employed to learn spatial correlations and local patterns across EEG channels by leveraging their strengths in localized spatial representation [16]. However, standard CNNs often lack the inherent capability to model long-range temporal dependencies effectively. To capture such temporal information, recurrent neural networks (RNNs) [17] and their advanced variants like long short-term memory (LSTM) units [18] are frequently used, although they can be susceptible to gradient vanishing issues. More recently, Transformer-derived architectures, particularly the Conformer model [19], have shown great promise by using self-attention to capture global temporal dependencies and have achieved significant success in sequential tasks. Despite their power, these models often come with substantial computational complexity and do not inherently integrate spatial or multi-modal feature processing.

In addition to the application of single features or single models, the integration of multiple information sources and attention mechanisms has become an important direction for improving EEG emotion recognition performance [20]. Attention mechanisms have further enhanced deep learning models by allowing them to focus on the most salient information. For instance, the CBAM [21] can refine feature maps by adaptively emphasizing important channels and spatial regions. In parallel, cross-modal fusion has emerged as a key strategy to leverage complementary information from different EEG characteristics, which typically encompass multiple modalities such as DE features that reflect the brain’s complex dynamics, and PSD features that describe the frequency-domain energy distribution. For example, Zhao et al. demonstrated the promise of this approach by using an MCA mechanism to effectively fuse DE and PSD features [22]. Despite this progress, a critical gap remains in the literature, as few works have successfully integrated powerful temporal modeling, fine-grained spatial-channel refinement, and synergistic cross-modal fusion into a single, unified framework. This limitation is evident even in the works closest to our approach, namely the EEG Conformer and the model proposed by Zhao et al. [22]. Our proposed TANet is distinct from both in its architectural synergy. While EEG Conformer effectively models temporal sequences, it lacks mechanisms for cross-modal feature fusion or dedicated spatial channel attention. Conversely, while Zhao et al. [22]. utilize MCA for feature fusion, their model relies on a 3D-CNN for spatiotemporal processing, whereas TANet employs the more powerful Conformer architecture to capture complex long-range temporal dependencies. By uniquely integrating these three complementary components—temporal modeling, spatial-channel attention, and cross-modal fusion—TANet provides a more comprehensive approach to feature extraction. Building on this architectural innovation, we also emphasize methodological transparency. While our experimental design adopts established paradigms for fair comparison with prior work, as detailed in the Methods section, we fully acknowledge the critical importance of robust, leakage-free evaluation protocols (e.g., subject-independent validation) that represent the gold standard in the field.

To overcome these limitations and raise the effectiveness of EEG emotion recognition, we present in this work an innovative triple-attention fusion network incorporating Conformer, CBAM, and MCA. The specific contributions of this study can be summarized as follows:

(1) We propose the TANet triple attention fusion network, which ingeniously integrates the powerful temporal modeling capabilities of Conformer, the spatial-channel attention enhancement mechanism of CBAM, and the cross-modal feature fusion capabilities of MCA into a unified framework, aiming to comprehensively and efficiently extract discriminative emotional features from EEG signals.
(2) We design a lightweight MCA module specifically to fuse DE and PSD features across various EEG frequency bands. This approach effectively captures complementary information from the two modalities, thereby generating a richer feature representation for subsequent temporal and spatial analysis.
(3) The proposed model demonstrates state-of-the-art performance on two widely recognized datasets, DEAP and SEED, under distinct evaluation protocols. The model attained an average accuracy of 98.51 ± 1.40% on the SEED dataset using a robust subject-specific 5-fold cross-validation protocol. On the DEAP dataset, to ensure a fair comparison with prior state-of-the-art works that focused on fine-grained pattern recognition, we adopted a similar segment-level random splitting paradigm. Under this specific setting, our model achieved mean accuracy of 99.69 ± 0.15% and 99.67 ± 0.13% for the valence and arousal dimensions, respectively, over 10 independent runs. We explicitly note that this evaluation context tests within-trial pattern discrimination rather than cross-subject generalization.
(4) The model in this paper exhibits fast convergence during the training process, and the accuracy in the validation phase quickly stabilizes, effectively mitigating the risk of overfitting and significantly reducing the training time. This high efficiency provides an efficient solution for the model in practical applications with limited computational resources or high real-time requirements.

The remainder of this paper proceeds as follows: Section 2 elaborates on our proposed method. Section 3 reports our experimental results, which are then discussed in Section 4, before the paper concludes in Section 5.

## 2. Materials and Methods

In this paper, we propose an innovative triple-attention fusion network TANet based on Conformer, CBAM and MCA, aiming to achieve high-performance emotion recognition by fusing temporal modeling, spatial channel attention and cross-modal feature fusion. This chapter will detail its overall architecture, the design principles of its core modules, the data preprocessing workflow, and the specific experimental setup.

### 2.1. Dataset Description

To validate the proposed model’s performance and generalization capabilities, we utilized two benchmark datasets for emotion recognition: DEAP and SEED.

The DEAP dataset, first introduced by Koelstra et al., is a multimodal resource containing EEG signals [12]. For its creation, 32-channel EEG data were collected from 32 individuals as they viewed 40 one-minute-long music videos. While the original dataset includes annotations for valence, arousal, dominance, and likability, our work concentrates on the binary classification of the valence and arousal dimensions. This results in 1280 samples per dimension. For our model input, we extracted both DE and PSD features across five distinct frequency bands, creating a comprehensive multi-modal feature representation. A summary of the DEAP dataset’s specifications can be found in Table 1.

The second dataset, SEED, was developed by Zheng et al. at Shanghai Jiao Tong University [13]. This dataset features EEG signals from 15 participants who were exposed to emotionally evocative stimuli. Over three separate sessions, each individual watched 15 movie clips designed to elicit positive, neutral, or negative emotional states. A key advantage of the SEED dataset is that it offers pre-extracted DE and PSD features. For each trial, these features are provided in a (5, 62, 10) tensor, which corresponds to the five frequency bands, 62 channels, and 10 time windows. Further details are summarized in Table 2.

The distinct data collection protocols, feature types, and emotional stimuli of the DEAP and SEED datasets collectively offer a robust framework for assessing the performance and generalizability of our proposed model.

### 2.2. Data Preprocessing

To ensure that the model can efficiently process EEG signal features from the SEED and DEAP datasets, this study designed a targeted data preprocessing workflow to accommodate the differences between the two datasets in terms of channel count, time dimension, and task type. For the SEED dataset, preprocessing begins with loading preprocessed DE and PSD features, whose feature shape is (number of samples, 5, 62, 10), representing 5 frequency bands, 62 channels, and 10-time windows, respectively. The data is first normalized through standardization operations. For the DE features, we applied the Standard Scaler method to each frequency band, transforming the data to have a zero meaning and unit variance. The PSD features underwent a two-step process: they were first scaled using a square root transformation to mitigate differences in their numerical ranges, and were subsequently standardized with the same Standard Scaler method. This processing approach effectively reduces numerical fluctuations in the features, enhancing the stability of model training. Crucially, to prevent any information leakage from the validation set into the training process, this standardization was performed independently for each fold of the cross-validation. Within each fold, the StandardScaler was fitted only on the training data for that fold and was subsequently used to transform both the training and the validation sets. This ensures that the parameters of the scaler (mean and standard deviation) were computed without any knowledge of the validation data. For the SEED dataset, we employ a subject-specific evaluation protocol. For each of the 15 participants, their data is independently partitioned using a 5-fold cross-validation scheme. This ensures that for each subject, the model is trained and validated on distinct subsets of their own data, allowing for a robust assessment of intra-subject performance. The final reported metrics, including accuracy, F1-score, and Kappa, are presented as the mean and standard deviation averaged across all 15 subjects, providing a comprehensive view of the model’s overall stability and performance. For each subject, data from all three experimental sessions were concatenated prior to this partitioning to create a comprehensive intra-subject dataset.

For the DEAP dataset, data preprocessing requires handling a more complex raw feature structure, with the initial shapes of the DE and PSD features being (1280, 32, 5, 58, 4), representing 1280 samples, 32 channels, 5 frequency bands, a 58 s time series, and 4-time segments, respectively. First, by averaging the last 4 s of data, the time dimension is extended from 58 s to 60 s, forming a feature tensor with the shape (1280, 32, 5, 60) to unify the time resolution. Subsequently, we employed the MCA module to fuse the DE and PSD features across each frequency band, which produced fused features of a uniform shape. This fusion process fully utilizes the complementary information between DE and PSD, providing richer emotional expressions for subsequent feature extraction. The fused features are then subjected to Min-Max Scaling to compress their numerical range to the [0, 1] interval, further enhancing model training efficiency. To bolster the model’s ability to capture temporal dynamics, we first partitioned the temporal dimension of the fused features into 3 s intervals. This process created a sliding window with a segment length of 3, ultimately yielding a feature tensor with the dimensions (25,600, 32, 5, 3). In terms of label processing, the valence and arousal scores are binarized separately (values below 5 are 0, and values of 5 or above are 1), and each label is repeated 20 times according to the time segmentation rules to match the number of feature segments, resulting in a final label dimension of (25,600). After binarization, the dataset’s class balance was assessed. For the valence dimension, the split was 41.1% for the low class and 58.9% for the high class. For the arousal dimension, the split was 43.4% for the low class and 56.6% for the high class. This indicates the absence of a severe class imbalance that would compromise the validity of the results. In the final step, we aggregated the 25,600 segments from all participants and partitioned them into training and testing sets via stratified sampling with a 9:1 ratio. Our choice of this entire “slice-first, then divide” workflow, including the holistic normalization performed on the aggregated data, was a deliberate decision to ensure a direct and fair benchmark against a series of high-performance models, such as the influential work by Zhao et al., which utilized the exact same workflow [22]. This approach allows us to isolate and rigorously validate the superior feature extraction capabilities of our proposed TANet architecture. While this protocol is designed to assess fine-grained, within-trial pattern recognition rather than cross-subject generalization, it serves as a crucial benchmark to demonstrate that TANet significantly advances the state-of-the-art under these established competitive conditions. To ensure the robustness and reliability of our findings, this entire process of data partitioning, model training, and evaluation was repeated 10 times using different random seeds. The final reported results are the mean and standard deviation of these 10 independent runs.

### 2.3. Feature Extraction

For our feature extraction pipeline, we focused on two powerful frequency-domain metrics derived from the preprocessed EEG signals: DE and PSD. We selected these features because they provide complementary insights into the brain’s emotional states, respectively, reflecting the signal’s complexity and its energy distribution. To effectively isolate neural activity associated with specific emotions, this feature extraction was applied independently to each of the five conventional EEG frequency bands: Delta, Theta, Alpha, Beta, and Gamma.

#### 2.3.1. Differential Entropy Extraction

Differential entropy, a key metric from information theory, serves to measure the uncertainty of continuous random variables. In the context of EEG signal analysis, it is widely utilized to quantify the complexity or activation level of brain activity within specific frequency bands. When an EEG signal is processed by filtering it for a specific frequency band within a certain time window, its distribution can be approximated as Gaussian, denoted by $\begin{array}{c} N(\mu ,\sigma^{2}) \end{array}$. For any random variable X that follows a Gaussian distribution with a mean of $\begin{array}{c} \mu \end{array}$ and a variance of $\begin{array}{c} \sigma^{2} \end{array}$, its probability density function is given by Equation (1):

(1) $$\begin{array}{c} f(x)=\frac{1}{\sqrt{2\pi \sigma^{2}}}e^{-\frac{{\left(x-\mu \right)}^{2}}{2\sigma^{2}}} \end{array}$$

Its differential entropy is defined as Equation (2):

(2) $$\begin{array}{c} h(X)=-\int_{-\infty }^{\infty }f(x)\log f(x)dx=\frac{1}{2}\log(2\pi e\sigma^{2}) \end{array}$$

Among them, $\begin{array}{c} \sigma^{2} \end{array}$ represents the variance of the EEG signal in the specific frequency band within the analysis time window. The DE feature is calculated by computing the variance of the EEG signal after filtering each channel in the sliding window at various frequency bands. This method can effectively capture the nonlinear characteristics of the EEG signal and reflect the complex dynamic changes in the brain in different emotional states.

#### 2.3.2. Power Spectral Density Extraction

To analyze the rhythmic patterns of brain activity, we utilize PSD, a standard method that measures how an EEG signal’s energy is allocated across the frequency spectrum. Our work specifically employs Welch’s method for this PSD calculation, a technique known for producing a more stable and reliable spectral estimate with lower variance. This method operates by segmenting a long signal into overlapping portions, applying a window function and a fast Fourier transform to each, and then averaging their resulting power spectra. The formula for the kth PSD value at a given frequency f is presented in Equation (3):

(3) $$\begin{array}{c} p_{k}(f)=\frac{1}{W}|F_{k}(f){|}^{2} \end{array}$$

Within this equation, the parameters are defined as follows: W represents the weight of the Hanning window, $F_{k}(f)$ is the fast Fourier transform of a 128 Hz windowed signal, and the window duration is 2 s. The overall PSD estimate is then calculated by aggregating the results from all segments, a process detailed in Equation (4):

(4) $$\begin{array}{c} P_{s}(f)=\frac{1}{K}\sum_{k=1}^{K}p_{k}(f) \end{array}$$

Among them, K is the total number of signal segments. PSD is divided into five frequency bands to generate shapes consistent with DE. This frequency band division method helps to avoid information crossover and improve feature discrimination. Finally, DE and PSD features are organized into multi-channel, multi-frequency band, and multi-time window tensors as raw feature representations for subsequent model input.

### 2.4. Model Architecture

**Figure 1:**
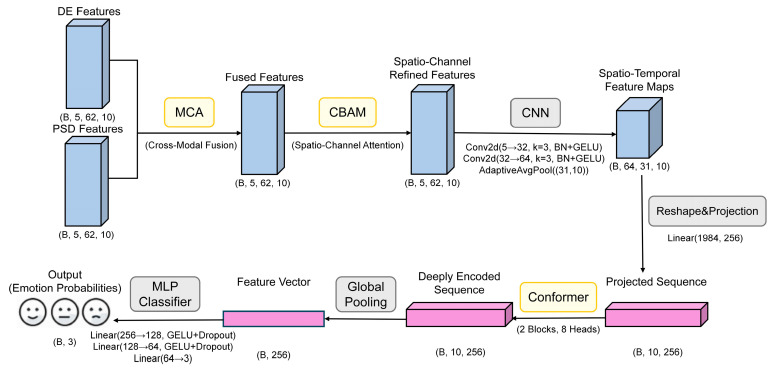
The TANet network model architecture proposed in this paper.

To realize efficient EEG emotion recognition, this paper proposes a triple-attention deep network TANet fusing Conformer, CBAM and MCA. This network fully integrates the temporal features of EEG signals, spatial-channel information, and the semantic complementarity between multimodal features. The overall model consists of five main modules: cross-modal attention module, CBAM spatial-channel attention module, convolutional feature extraction module, Conformer temporal modeling module, and final classifier. The model workflow is shown in Figure 1.

As shown in Figure 1, the specific processing flow of the model is illustrated using the SEED dataset as an example. Initially, the input DE and PSD features undergo cross-modal fusion via the MCA module to create a unified multimodal representation. This representation is then spatially and spectrally refined by the CBAM, which accentuates critical channels and regions. Following this, a convolutional module compresses these features before they are passed to the Conformer module, a component tasked with modeling both local and global temporal dynamics. The final step involves a classifier that determines the ultimate emotion category. This multi-stage architecture is designed to comprehensively exploit the multidimensional characteristics of EEG signals, thereby enhancing classification performance.

For a granular, layer-by-layer breakdown of this architecture, Table 3 details the precise dimensional transformations a single sample undergoes. This table uses a single sample from the SEED dataset as an example to describe the complete dimensional transformation process from input to output. Within the table, the notations B, F, C, and T correspond to batch size, number of frequency bands, number of EEG channels, and number of time windows, respectively. The complete TANet architecture has a total of 3.34 million trainable parameters. For a single forward pass, the model requires approximately 1.90 GFLOPs and achieves an average inference time of 6.15 ms per sample on an NVIDIA RTX 3080 Ti GPU.

As shown in Table 3, the model first effectively fuses the DE and PSD features through the MCA module for each frequency band and refines the spatiotemporal features through the CBAM. Subsequently, two layers of 2D convolutional networks and adaptive pooling layers are responsible for extracting and integrating local patterns from multi-frequency feature maps, efficiently converting the feature map dimensions from (B, 5, 62, 10) to (B, 64, 31, 10). After dimension rearrangement and linear projection, the data is converted into a sequence of length 10 and dimension 256 (B, 10, 256) and fed into two stacked Conformer modules for in-depth temporal dependency modeling. Finally, through global average pooling and a three-layer fully connected classifier, the model outputs the predicted probabilities corresponding to the three emotion categories. The entire process achieves a stepwise transformation from multi-modal, multi-channel raw features to highly abstract classification features.

To provide a clear, step-by-step overview of the model’s data flow, the procedural workflow of the proposed TANet architecture is summarized in Algorithm 1 The algorithm begins with cross-modal fusion of DE and PSD features at the frequency band level. The resulting fused features then undergo spatial-channel refinement via the CBAM. Subsequently, local patterns are extracted by a CNN block, and the features are projected into a sequence format suitable for the Conformer. Finally, the Conformer modules capture temporal dependencies, and an MLP classifier produces the final emotion probabilities.


**Algorithm 1.** Pseudocode for TANet Emotion Recognition FrameworkInput: DE features (B, F, C, T), PSD features (B, F, C, T)Output: Emotion category probabilities1. For each frequency band f in F:2. fused_features[f] = MCA(DE[f], PSD[f]) -- Mutual Cross-modal fusion3. stacked_fused = Stack (fused_features along frequency dimension) -- Shape: (B, F, C, T)4. refined_features = CBAM (stacked_fused) -- Spatial-channel refinement5. conv_features = CNNBlock (refined_features) -- Conv2D + BN + GELU + Pool, local feature extraction6. projected_seq = Project (conv_features) -- Reshape and project to sequence, Shape: (B, T, D)7. temporal_features = Conformer (projected_seq) -- Temporal modeling, using two Conformer blocks8. pooled_features = GlobalAvgPool (temporal_features)9. probabilities = Classifier (pooled_features) -- MLP classifier with softmax activation Return probabilities


#### 2.4.1. Cross-Modal Attention Fusion Module

The objective of the MCA module is to fuse features from the DE and PSD modalities to capture the complementary information between them. For the SEED experiment, the MCA module accepts both DE and PSD features as input, each with a shape of (B, 62, 10), where B is the batch size, 62 is the channel count, and 10 represents the number of time slices. The module operates by first generating queries, keys, and values from these inputs via linear transformations. These are then used to compute the final output using the standard attention mechanism, as detailed in Equation (5):

(5) $$\begin{array}{c} Attention(Q,K,V)=soft\max\left(\frac{QK^{⊤}}{\sqrt{d_{k}}}\right)V \end{array}$$

Among them, $d_{k}$ is the attention dimension. The output dimension remains unchanged, and attention fusion is finally performed and stacked on five frequency bands, forming a fusion feature tensor with the shape (B, 5, 62, 10). Dropout is used in this process to enhance regularity [23] and reduce overfitting.

**Figure 2:**
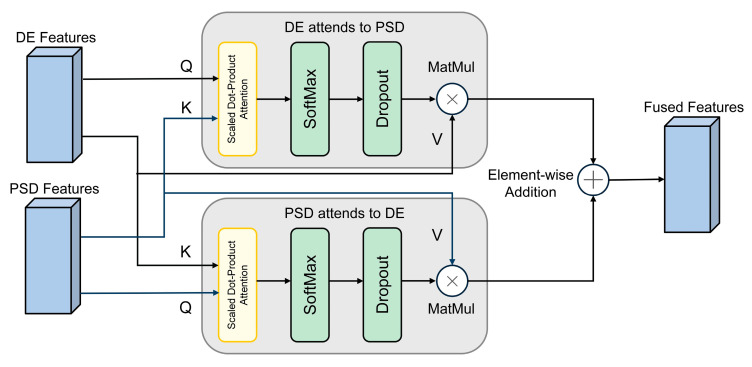
Schematic structure of the MCA module used in the DEAP experiments. Q, K, and V are the queries, keys, and values in the attention mechanism.

In the DEAP experiment, in order to more fully integrate DE and PSD information, the MCA module implements a bidirectional cross-attention mechanism, the detailed structure of which is shown in Figure 2. This mechanism allows the features of the two modalities to query and enhance each other. Specifically, the DE and PSD features, with initial shapes of (B, 32, 60), participate in the interaction as queries and keys, respectively. The fusion process can be defined by Equation (6):

(6) $$\begin{array}{c} MCA(f_{1}\;,f_{2})=Atten(f_{1}\;,f_{2}\;,f_{2})+Atten(f_{2}\;,f_{1}\;,f_{1}\;) \end{array}$$

where $f_{1}$ and $f_{2}$ represent the DE and PSD modal features, respectively. The fusion output is (B, 32, 60) and is stacked in the frequency dimension to form a complete tensor (B, 32, 5, 60). Dropout is set to 0.3 to further improve generalization ability.

#### 2.4.2. CBAM Spatial Channel Attention Module

**Figure 3:**
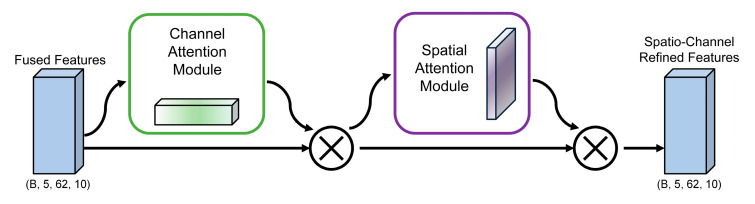
The illustration of the CBAM’s architecture.

Within our TANet architecture, the CBAM Spatial-Channel Attention Module is tasked with refining the fused feature maps passed from the MCA layer. Its primary goal is to adaptively select and emphasize the most salient channel-wise and spatial features that are critical for emotion recognition. As illustrated in Figure 3, this refinement is accomplished through a sequential, two-step attention process. The module first applies channel attention across the feature map to assign higher importance to specific frequency bands or channels most indicative of an emotional state. Subsequently, the resulting channel-refined feature map undergoes spatial attention, where the model learns to focus on key spatial locations that likely correspond to relevant brain regions. By successively applying these two attention mechanisms, the CBAM generates a doubly refined feature map that is then passed to the next stage of the network. The detailed computational formulas for both channel and spatial attention are described below.

For the input feature map $F\in {\mathbb{R}}^{C\times H\times W}$ (where C is the number of channels, H is the height, and W is the width), the channel attention module aims to generate a 1D channel attention weight vector. It first performs global average pooling and global max pooling on F, yielding $\begin{array}{c} F_{avg}^{c}\in {\mathbb{R}}^{C\times 1\times 1} \end{array}$ and $\begin{array}{c} F_{max}^{c}\in {\mathbb{R}}^{C\times 1\times 1} \end{array}$, respectively. These aggregated features are processed through a shared two-layer perception, and the results are then summed elementwise and passed through a Sigmoid activation function to generate the channel attention weights $\begin{array}{c} \begin{array}{c} M_{c}(F) \end{array}\in {\mathbb{R}}^{C\times 1\times 1} \end{array}$. The formula for calculating the channel attention $\begin{array}{c} M_{c}(F) \end{array}$ is shown in Equation (7):

(7) $$M_{c}(F)=\sigma (MLP(AvgPool(F))+MLP(MaxPool(F)))$$

Here, $\begin{array}{c} \sigma \end{array}$ denotes the sigmoid activation function. Subsequently, the original feature map is multiplied by the channel attention weights, and the channel-enhanced features $F^{\prime }\in {\mathbb{R}}^{C\times H\times W}$ are obtained through element-wise multiplication ⊗.

This refined feature map $F^{\prime }$ subsequently serves as the input for the spatial attention module, which generates a 2D spatial attention map. Its process begins by pooling features along the channel axis using both average and max pooling to create two maps: $F_{avg}^{s}\in {\mathbb{R}}^{1\times H\times W}$ and $F_{max}^{s}\in {\mathbb{R}}^{1\times H\times W}$. These two feature maps are first concatenated. The resulting combined map is then passed through a convolutional layer, and a Sigmoid activation function is subsequently applied to the output. This process yields the final spatial attention weights, denoted as $M_{s}(F^{\prime })\in {\mathbb{R}}^{1\times H\times W}$. The exact formula for this calculation is detailed in Equation (8):

(8) $$M_{s}(F^{\prime })=\sigma (Conv(Concat(AvgPool(F^{\prime }),MaxPool(F^{\prime })))))$$

The final enhanced feature $F^{\prime \prime }$ is obtained by multiplying the channel-enhanced feature $F^{\prime }$ by the spatial attention weight as shown in Equation (9):

(9) $$F^{\prime \prime }=F^{\prime }⊗M_{s}(F^{\prime })$$

In the SEED and DEAP experiments, CBAM receives the (B, 5, 62, 10) feature tensor output from MCA and performs attention guidance and feature enhancement for each channel and time window dimension of the frequency band. This module can significantly improve the discriminability of features and effectively suppress background noise interference.

#### 2.4.3. Convolution Feature Extraction Module

The convolution feature extraction module is responsible for extracting local patterns in space and time from EEG feature maps. In both the SEED and DEAP experiments, this module employs two layers of 2D convolution layers, each followed by batch normalization [24] and a GELU activation function [25]. The first layer maps the five input frequency band channels to 32 output channels, while the second layer further increases the number of channels to 64. Subsequently, an adaptive average pooling layer fixes the feature dimensions to (64, 31, 10), ensuring consistency in the input dimensions received by the Conformer module. The output features from the convolutional stage are first reshaped and subsequently projected into a 256-dimensional space to serve as the input sequence for the Conformer module. This design choice enables the model to effectively capture local spatial and temporal patterns within the EEG signals across different channels and time windows.

The calculation process for two-dimensional convolution operations is as shown in Equation (10):

(10) $$Y_{i,j}^{\left(k\right)}=\sum_{c=1}^{C_{\ln}}\sum_{m=1}^{h}\sum_{n=1}^{w}W_{m,n}^{\left(k,c\right)}\cdot X_{i+m,j+n}^{\left(c\right)}+b^{\left(k\right)}$$

Among them, $X_{i+m,j+n}^{\left(c\right)}$ is the input feature map, $Y^{\left(k\right)}$ is the feature map of the kth output channel, $W^{\left(k,c\right)}$ is the convolution kernel from the cth input channel to the kth output channel, $b^{\left(k\right)}$ is the bias of the kth output channel, and i,j is the spatial position index.

#### 2.4.4. Conformer Timing Modeling Module

**Figure 4:**
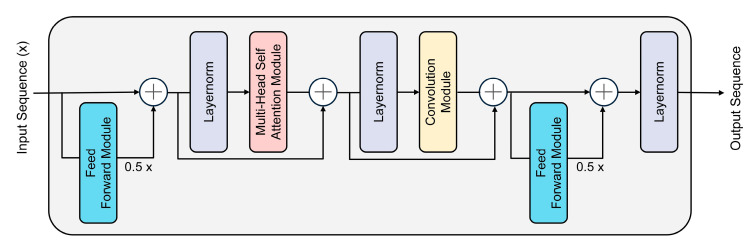
The illustration of the Conformer module’s internal architecture.

The Conformer module is $X\in {\mathbb{R}}^{C_{in}\times H\times W}$ the core component of this model, responsible for modeling the complex temporal dependencies of EEG signals. The internal architecture is composed of four primary submodules: a feedforward module, a multi-head self-attention module, a convolution module, and a residual connection. Figure 4 intuitively shows the detailed data processing flow of a single Conformer module.

As depicted in Figure 4, the input sequence first passes through a feedforward module with a scaling factor of 0.5 for the first feature transformation. Subsequently, the data stream sequentially passes through a multi-head self-attention module and a convolutional module to capture the long-range global dependencies and local correlations of the sequence, respectively. It is worth noting that layer normalization is applied before each core module, and the outputs are added to the inputs via residual connections to ensure stable information flow and effective gradient propagation. After passing through the second half-step feedforward module, the final output is processed by a layer normalization layer. This structural design enables Conformer to demonstrate outstanding performance in temporal modeling tasks. Next, we will provide a detailed explanation of the design principles of each submodule within the architecture.

The feedforward module uses two layers of linear transformation and GELU activation, combined with Dropout to prevent overfitting. For an input tensor denoted as $x\in {\mathbb{R}}^{B\times D\times T}$, where B, T and D represent the batch size, sequence length, and feature dimension, respectively, the entire calculation is formulated in Equation (11):

(11) $$FFN(x)=Dropout(Linear_{2}(GELU(Linear_{1}(LayerNorm(x))))))$$

Multi-head self-attention uses eight attention heads to model global dependencies in parallel and capture feature associations in different subspaces. The convolution module uses depth-separable convolution in conjunction with BatchNorm and GELU to capture local sequence patterns. The depth-separable convolution operation itself is a two-step process, consisting of a depthwise convolution followed by a pointwise convolution. The mathematical formula for the initial depthwise convolution step is provided in Equation (12):

(12) $$Z_{t}^{\left(c\right)}=\sum_{k=-K}^{K}w_{k}^{\left(c\right)}\cdot X_{t+k}^{\left(c\right)}$$

Among them, $Z_{t}^{\left(c\right)}$ represents the output of the cth channel at time step, $w_{k}^{\left(c\right)}\in \mathbb{R}$ is the one-dimensional convolution kernel on the cth channel, K is half the length of the convolution kernel, and $X_{t+k}^{\left(c\right)}$ is the input sequence of the cth channel. The formula for pointwise convolution is Equation (13):

(13) $$Y_{t}^{\left(j\right)}=\sum_{c=1}^{C}p^{\left(j,c\right)}\cdot Z_{t}^{\left(c\right)}$$

Among them, $p^{\left(j,c\right)}\in \mathbb{R}$ is the pointwise convolution weight from channel c to output channel j, and $Y_{t}^{\left(j\right)}$ is the final output of the jth output channel at time step t. Residual connections are employed to sum the outputs of all submodules with the original input. Following this operation, layer normalization is applied to facilitate smooth gradient propagation and promote stable model convergence.

In the SEED and DEAP experiments, the Conformer module receives (B, 10, 256) sequence features projected from the convolutional feature extraction layer, i.e., feature vectors containing 10 steps, each with 256 dimensions. These sequence features are processed through two stacked Conformer blocks, followed by global average pooling to aggregate the temporal dimension, resulting in the final (B, 256) vector used for classification. This module effectively integrates local details with global trends, enabling precise modeling of EEG temporal dynamics.

#### 2.4.5. Classifier Design

The classifier receives the output features from the Conformer module and performs the final emotion classification task. For the SEED experiment, the classifier is constructed from a three-layer fully connected network, which incorporates both GELU activation and Dropout layers. This classifier is responsible for producing outputs across three emotional categories: positive, neutral, and negative. In the DEAP experiment, the classifier structure is consistent with the SEED version, but the output layer maps two categories of emotions: valence and arousal. The classifier design fully considers the output requirements of different tasks, ensuring efficient emotion classification performance.

### 2.5. Loss Function and Optimization Strategy

To effectively train the TANet triple attention network proposed in this paper, we carefully designed the loss function and optimization strategy to ensure that the model has good learning ability and generalization performance.

This study uses a weighted cross-entropy loss function to effectively measure the difference between the model’s predicted probability and the true label, which is suitable for the binary classification task of DEAP and the ternary classification task of SEED. Its mathematical expression is Equation (14):

(14) $$\begin{array}{c} L=-\overset{C}{\underset{i=1}{\sum }}w_{i}\left[(1-\epsilon )y_{i}+\frac{\epsilon }{C}\right]\log({\hat{y}}_{i}) \end{array}$$

Among them, C denotes the number of categories, $y_{i}$ is the true label, ${\hat{y}}_{i}$ represents the model’s the predicted probability, $w_{i}$ is the category weight, and ϵ stands for the label smoothing coefficient.

To address the significant class imbalance present in the DEAP dataset’s valence and arousal dimensions, we implemented a class weighting scheme during the training process. This scheme assigned weights to each class that were inversely proportional to the number of samples in that class, compelling the model to pay greater attention to minority classes and thus mitigating bias toward majority classes. Furthermore, to enhance the model’s robustness and generalization, we incorporated a label smoothing mechanism into the loss function. This technique converts hard labels into soft probabilistic distributions, which helps to alleviate the model’s tendency to overfit to specific training examples. To ensure methodological rigor, these hyperparameters were not tuned on the validation sets. Instead, the class weights were dynamically calculated for each training fold based on its specific class distribution, and a fixed label smoothing coefficient of 0.1, as indicated in the loss function, was used across all experiments based on common practice. This approach preserves the validation set as an unbiased tool for evaluating generalization performance.

The optimizer selected was AdamW [26], which improves generalization ability by decoupling weight decay. Compared to the traditional Adam optimizer, it generally performs better in terms of model generalization ability. Learning rate scheduling is critical to the training process. This study adopted differentiated learning rate scheduling strategies tailored to the characteristics of different datasets. When processing the DEAP dataset, we tended to use schedulers that smoothly decrease the learning rate to promote stable convergence. When handling the complex cross-subject validation scenarios of the SEED dataset, we selected schedulers that dynamically adjust the learning rate to accelerate convergence.

To fully leverage GPU computational power and improve training efficiency, this study employs mixed-precision training during the training process, i.e., while maintaining FP32 (single-precision floating-point) weight copies, most computations are performed using FP16 (half-precision floating-point), significantly accelerating training and reducing memory usage. To properly handle gradient scaling issues that may arise from half-precision training, we integrate the gradient scalers provided by PyTorch (version 2.7.1). Additionally, to effectively train larger batches of models under limited GPU memory conditions while ensuring training stability, we introduced a gradient accumulation mechanism. To prevent gradient explosion during training, we also applied gradient clipping to model parameters. The implementation of these comprehensive optimization strategies aims to ensure the stability and efficiency of model training, laying the foundation for achieving high performance.

## 3. Results

This chapter focuses on the empirical evaluation of the proposed TANet network for EEG-based emotion recognition, covering our experimental design, evaluation metrics, and final results. To validate the model’s effectiveness and robustness, our analysis incorporates three main components. First, a comparative study benchmarks TANet’s performance against leading state-of-the-art methods. Second, ablation studies are conducted to isolate and quantify the specific contribution of each module within the model. Finally, visualization techniques are employed to analyze the feature distributions learned by TANet, offering an intuitive understanding of its internal mechanisms.

### 3.1. Experimental Setup

#### 3.1.1. Metrics for Performance Evaluation

For a thorough performance assessment of the TANet model on EEG emotion recognition tasks, we employed three standard evaluation metrics: accuracy, the F1 score [27], and the Kappa coefficient [28]. These indicators quantify the model’s performance by, respectively, measuring its correctness, classification balance, and consistency. These metrics were applied according to the specifics of each dataset: for the SEED dataset, they evaluated the three-class classification task, while for the DEAP dataset, they were applied to the binary classification task.

Among these, Accuracy provides a direct measure of overall correctness, calculated as the fraction of all samples that the model classified correctly. The formal definition is provided in Equation (15):

(15) $$\begin{array}{c} Acc=\frac{TP+TN}{TP+TN+FP+FN}\; \end{array}$$

Among them, $\begin{array}{c} TP\; \end{array}$ and TN represent the number of correctly identified positive samples and negative samples, respectively, while $\begin{array}{c} FP\; \end{array}$ and FN represent the number of incorrectly identified positive samples and negative samples, respectively.

The F1 score synthesizes precision and recall into a single metric via their harmonic meaning. This approach is particularly robust for evaluating model performance on datasets with class imbalances. The calculation is specified in Equation (16):

(16) $$\begin{array}{c} F1=2\times \frac{Precision\times Recall}{Precision+Recall}=\frac{TP}{TP+\frac{1}{2}(FP+FN)}\; \end{array}$$

To gain a nuanced view of classification performance, we utilize the complementary metrics of Precision and Recall. Precision assesses the exactness of the model’s positive predictions, indicating how many selected items were truly relevant. Recall, on the other hand, assesses the model’s completeness, indicating how many of all relevant items were successfully identified. Because a trade-off often exists between these two, the F1 score is employed to synthesize them into a single, balanced measure as their harmonic meaning. This is particularly valuable for the binary classification tasks on the DEAP dataset.

Furthermore, to ensure performance is not merely due to random chance, the Kappa coefficient is used. This metric provides a more robust measure of true predictive skill by explicitly correcting for the possibility of chance agreement. The formal calculation is outlined in Equation (17):

(17) $$\begin{array}{c} Kappa=\frac{P_{0}-P_{e}}{1-P_{e}}\; \end{array}$$

Among them, $P_{0}$ is the accuracy rate of actual classification, and $P_{e}$ is the accuracy rate of random classification. The Kappa coefficient is particularly important in cross-subject validation, as it can assess the robustness of the model when faced with individual differences.

#### 3.1.2. Training Process

The computational environment for this study was an NVIDIA RTX 3080 Ti GPU, with all models implemented in the PyTorch framework. Our optimization strategy involved using a weighted cross-entropy loss function, paired with label smoothing to enhance the model’s generalization capabilities. For the optimization algorithm, we selected AdamW and utilized its default momentum parameters (β_1_ = 0.9, β_2_ = 0.999). Hyperparameters were specifically tuned for each dataset. For the emotion recognition task on DEAP, we set the initial learning rate to 0.0005, weight decay to 1 × 10^−3^, and batch size to 32, with a CosineAnnealingLR scheduler for learning rate adjustments. In contrast, for the three-class task on the SEED dataset, the initial learning rate was 0.002, weight decay was 1 × 10^−4^, the batch size was 96, and the learning rate was optimized using a OneCycleLR scheduler.

The model is trained for 100 epochs on both datasets. To effectively prevent overfitting, we introduce an early stopping mechanism. To maximize the use of GPU computing power and improve training efficiency, we enable mixed-precision training in the SEED dataset experiments and use PyTorch’s torch.amp.GradScaler for gradient scaling. Additionally, to achieve a larger effective batch size under limited GPU memory and stabilize the training process, we adopted a gradient accumulation mechanism with a step count of 2. To further ensure training stability and prevent gradient explosion, we applied gradient clipping to the model parameters, limiting the maximum norm of the gradient to 1.0.

#### 3.1.3. Statistical Evaluation

To verify the statistical significance of performance improvements, comparisons between the proposed model and its ablated variants, as well as key benchmark models, were conducted. Depending on the data distribution, either paired *t*-tests or Wilcoxon signed-rank tests were performed on the results from all cross-validation folds for the SEED dataset and from repeated runs for the DEAP dataset. The 95% confidence interval (CI) of accuracy is also given. A *p*-value of less than 0.05 was considered statistically significant. All performance metrics are reported as mean ± standard deviation.

### 3.2. Ablation Experiment

This section aims to thoroughly evaluate the contribution of each key component in the proposed model to emotion recognition performance through a series of ablation experiments. We systematically remove or replace specific modules in the model to observe their impact on classification results, thereby quantifying the independent and synergistic effects of each component. Experiments leveraging the SEED and DEAP datasets were performed to systematically validate the model’s robustness and its ability to generalize.

#### 3.2.1. Ablation Experiments on the SEED Dataset

To assess the respective contributions of Conformer temporal modeling, CBAM spatial-channel attention, and MCA cross-modal attention modules, we designed four experimental sets on the SEED dataset. The best average accuracy for each module combination is presented in Table 4.

As demonstrated in Table 4, the full model incorporating Conformer, CBAM, and MCA delivered superior and more stable performance across all evaluation metrics. It achieved the highest mean accuracy of 98.51 ± 1.40%, alongside the best F1-score of 0.981 ± 0.015 and Kappa coefficient of 0.972 ± 0.021. This comprehensive performance significantly surpasses that of its ablated counterparts, an advantage confirmed to be statistically significant with a *p*-value below 0.001 for all comparisons. In contrast, removing any single module led to a clear decline in performance. The Conformer + CBAM combination, for instance, saw its accuracy drop to 97.35 ± 2.09%, and other ablated versions similarly showed lower metrics across the board. These results strongly demonstrate the synergistic effects of the combined modules, which collectively contribute not only to higher accuracy but also to a more robust and stable model performance.

#### 3.2.2. Ablation Experiments on the DEAP Dataset

We conducted a series of ablation studies to rigorously assess the contribution of each component to the overall robustness and generalization of our proposed emotion recognition model. These studies were performed using the DEAP dataset, with a specific focus on evaluating the core modules’ performance along with the arousal and valence dimensions. The results of this ablation analysis are presented in Table 5.

As shown in the results of Table 5, the proposed comprehensive model (Conformer + CBAM + MCA) demonstrated exceptional and stable performance across all metrics on the DEAP dataset. For the Arousal dimension, it achieved a mean accuracy of 99.67 ± 0.13%, an F1-score of 0.997 ± 0.001, and a Kappa coefficient of 0.993 ± 0.003. Similarly, for the Valence dimension, it recorded a mean accuracy of 99.69 ± 0.15%, an F1-score of 0.997 ± 0.002, and a Kappa of 0.993 ± 0.003. The model’s robustness across 10 independent runs is highlighted by the consistently low standard deviations and narrow 95% confidence intervals. A Wilcoxon signed-rank test confirmed this superior performance is statistically significant, with the full model outperforming all ablated variants (*p* < 0.001 for all comparisons). Removing any module led to a clear decline across all metrics. Most significantly, removing the Conformer temporal modeling module (CBAM + MCA) caused a drastic performance drop, with accuracies falling to 84.74% for Arousal and 84.10% for Valence, and Kappa coefficients plummeting to 0.707 and 0.697, respectively. This stark contrast highlights the critical role of Conformer in capturing temporal dependencies, as its absence leads to a much less effective and highly unstable model.

The ablation experiment results on the SEED and DEAP datasets are highly consistent, clearly indicating that the Conformer, CBAM, and MCA modules are all indispensable core components. Each plays a unique and critical role in temporal feature extraction, spatial-channel information enhancement, and cross-modal feature fusion. The statistical significance of these findings, validated with *p*-values consistently below 0.01 across both datasets, provides robust mathematical evidence for this conclusion. In particular, the Conformer module makes the most significant contribution to model performance, as its removal led to the most substantial decline in both mean accuracy and stability on both datasets. This emphasizes the central role of temporal modeling in EEG emotion recognition. Concurrently, MCA and CBAM further optimize feature representation by introducing effective attention mechanisms, synergistically enhancing the model’s overall performance and robustness. These results, now validated through repeated trials, fully confirm the effectiveness and superiority of the proposed triple-attention architecture.

### 3.3. Results on the SEED Dataset

In this portion of our study, we present an evaluation of the proposed TANet model’s performance on the SEED dataset. Our assessment methodology involves benchmarking the model against established approaches and is supplemented by visualization-based analyses. The SEED dataset, which provides the empirical basis for this evaluation, contains 62-channel EEG recordings from 15 individuals corresponding to three distinct emotional categories: positive, neutral, and negative. These signals have been decomposed into five standard frequency bands (Delta, Theta, Alpha, Beta, and Gamma). To ensure the statistical reliability and stability of our findings, we employ a 5-fold cross-validation scheme for the experiment.

#### 3.3.1. Comparison with the Benchmark Model

To validate the effectiveness of the TANet model on the SEED dataset, we compared its performance with several benchmark models: (1) SVM: A traditional machine learning algorithm that excels at handling small-scale data classification problems but it is limited in its capacity to extract the complex, nonlinear features inherent in EEG signals [29]; (2) 2D-CNN: A deep learning model based on time-frequency maps, which captures local spatial patterns through two-dimensional convolution operations and is widely used in pattern recognition tasks for EEG signals [30]; (3) DGCNN: A deep learning method based on graph convolutional networks, capable of effectively integrating the spatiotemporal dynamic information of EEG signals to enhance classification performance [31]; (4) EEGNet: A lightweight deep convolutional architecture specifically optimized for EEG signal classification tasks, balancing computational efficiency and classification accuracy [20]; (5) RGNN: A graph neural network method combining temporal dynamics and spatial structure, suitable for modeling the multidimensional feature relationships in EEG signals [32]; (6) DeepConvLSTM: A hybrid model that combines convolutional layers and long short-term memory units, aiming to simultaneously extract local spatial features and long-term temporal dependencies from EEG signals [33]; (7) TSception: A neural network architecture that uses multi-scale spatiotemporal convolutions, performing exceptionally well in EEG emotion recognition and particularly suited for processing complex temporal data [34]; (8) EEG Conformer: A hybrid model integrating convolutional operations and Transformer mechanisms, utilizing one-dimensional convolutions to extract local features and capturing global temporal associations through self-attention mechanisms, suitable for various EEG tasks including SEED [10]; (9) PGCN: A model based on a pyramid-based graph convolutional network, which aggregates features at local, mesoscopic, and global levels, combining neuroscientific knowledge to model the structural and functional connections between electrodes [35]. Evaluation metrics include accuracy, F1 score, and Kappa score to comprehensively assess the model’s classification performance. Experimental results are shown in Table 6.

As detailed in Table 6, our proposed TANet model demonstrated both high performance and excellent stability, achieving a mean accuracy of 98.51 ± 1.40%, a mean F1 score of 0.972 ± 0.021, and a mean Kappa coefficient of 0.981 ± 0.015 across all subjects on the SEED dataset. This performance significantly surpasses all baseline methods included in the comparison, underscoring the model’s advanced and robust capabilities for EEG emotion recognition. The advantage of our model over traditional machine learning and conventional CNN approaches lies in its deep learning architecture. This design allows it to extract high-level abstract features while also overcoming the inherent weakness of standard CNNs in modeling long-range temporal dependencies. Compared to models focused on graph structures or time series, the TANet demonstrates more significant performance improvements, primarily due to the introduction of the CBAM spatial-channel attention module and the MCA cross-modal attention module, which achieve deep refinement and fusion of spatial, channel, and cross-modal features. CBAM adaptively focuses on key regions, while MCA fully utilizes the complementary information from DE and PSD. Notably, even when compared to state-of-the-art hybrid models like EEG Conformer, TANet achieves a significant performance improvement of approximately 3.21%. This strongly demonstrates the unique value and powerful synergistic effects of the triple attention mechanism proposed in this paper, which comprehensively enhances the model’s feature extraction and discrimination capabilities through multi-dimensional information fusion, ultimately achieving higher emotion recognition accuracy.

To rigorously evaluate the contribution of each core component, we performed statistical analyses comparing our full TANet model against each of its ablated versions. As established in our ablation study results, statistical tests such as the paired *t*-test or Wilcoxon signed-rank test confirmed that the full model’s performance was significantly superior to all three ablated counterparts, with a *p*-value below 0.001 for all comparisons. This provides robust statistical evidence that the contributions of the Conformer, CBAM, and MCA modules are each individually significant, highlighting the effectiveness and synergistic design of our triple-attention architecture.

#### 3.3.2. Feature Distribution Visualization

**Figure 5:**
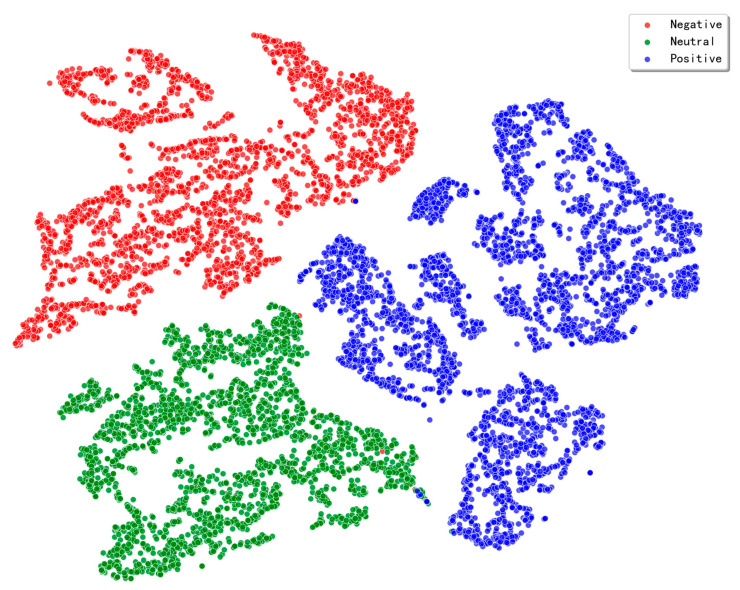
Visualization of t-SNE feature distribution of the TANet model for subject 1 in the SEED dataset. Different colors in the figure represent different emotion categories (red: negative, green: neutral, blue: positive).

To provide a clearer insight into the superior performance achieved by the TANet model on the SEED dataset, we conducted a visualization analysis of the final feature representations learned by the model. We employed t-SNE nonlinear dimensionality reduction technology to map the high-dimensional feature vectors extracted by the model before the classification layer into a two-dimensional space [36]. Taking subject 1 from the SEED dataset as an example, the feature distribution is plotted as shown in Figure 5.

As shown in Figure 5, the features learned by the TANet model exhibit extremely clear, compact, and distinctly bounded clusters in the two-dimensional space. Sample points from different emotional categories are highly separated, forming independent clusters with minimal overlap between categories, nearly achieving complete distinction. This intuitively demonstrates the TANet model’s robust feature extraction capability and high discriminative power for EEG emotional states. The model effectively captures subtle patterns related to emotions in EEG signals and maps them to a feature space with high separability.

#### 3.3.3. Confusion Matrix Analysis

**Figure 6:**
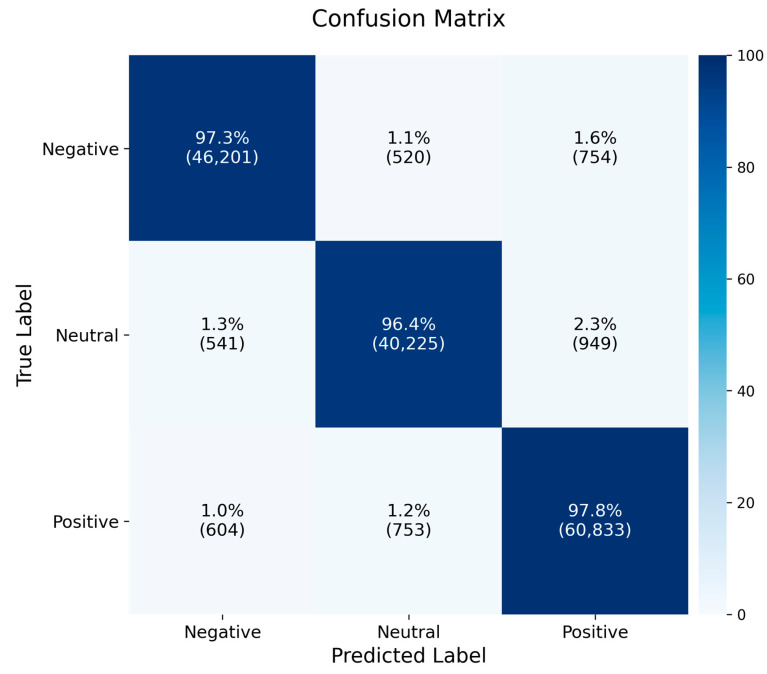
Normalized confusion matrix for the TANet model on the SEED dataset.

To provide a more detailed evaluation of the TANet model’s classification performance on the SEED dataset, we created a normalized confusion matrix, which is presented in Figure 6. This matrix is aggregated over all folds and all subjects to provide a comprehensive overview of the model’s classification behavior. This matrix visualizes the distribution of the model’s predictions across the positive, neutral, and negative emotion categories, presenting the values as percentages. The diagonal elements in the matrix reflect the model’s high accuracy for each category, while the off-diagonal elements are close to 0%, indicating an extremely low misclassification rate. This indicates that the model can effectively distinguish between different emotion categories, demonstrating strong discriminative capability.

#### 3.3.4. ROC Curve Analysis

**Figure 7:**
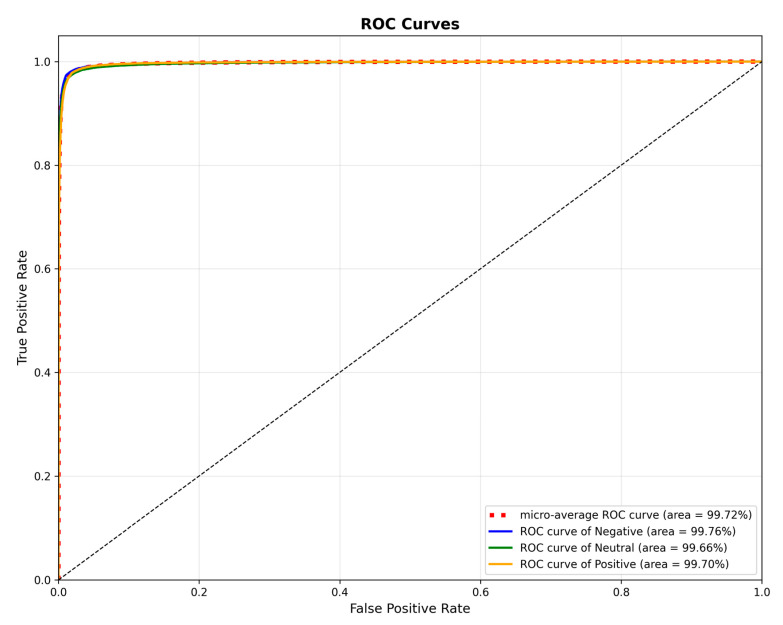
ROC curve of the TANet model on the SEED dataset.

To assess the classification performance of our model, we generated Receiver Operating Characteristic (ROC) curves for the SEED dataset, as presented in Figure 7. These curves illustrate the model’s performance on the positive, neutral, and negative categories, yielding individual Area Under the Curve (AUC) values of 99.76%, 99.66%, and 99.70%, respectively. Furthermore, a micro-averaged AUC of 99.72% was achieved, signifying the model’s exceptional overall discriminative capability in this multi-class task. These findings provide additional validation for the TANet model’s effectiveness at capturing emotional features from EEG signals.

#### 3.3.5. Spatial Attention Map Visualization Analysis

**Figure 8:**
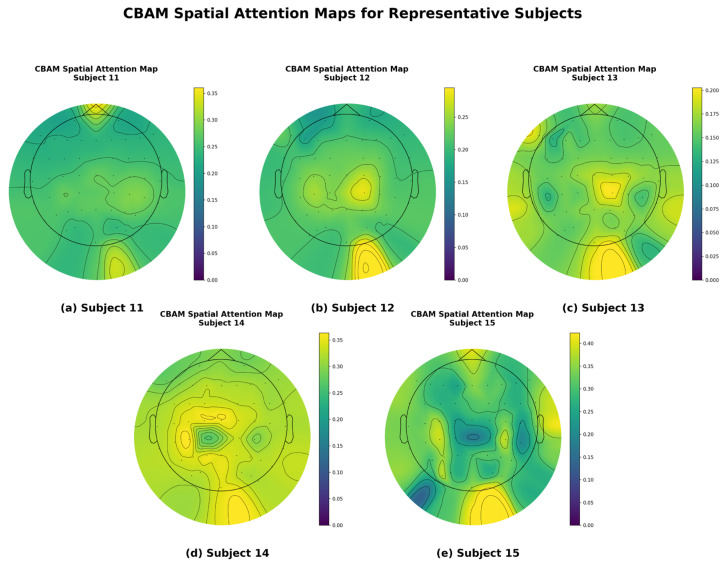
CBAM spatial attention maps for representative subjects in the upper part of the SEED dataset. (**a**–**e**) show the spatial attention weight distributions for subjects 11 to 15, respectively. The brighter areas (yellow) in the figure indicate that the model assigned higher attention weights to those spatial locations, suggesting that these brain regions are considered more important in the emotion recognition task.

To gain a more intuitive understanding of how this model enhances emotion recognition performance through spatial attention mechanisms, we visualized the EEG topographies of some participants, as shown in Figure 8. To ensure the representativeness and scientific validity of the visualization results, the five participants shown in the figure were carefully selected, with their respective classification accuracy rates covering high, medium, and low performance levels. This selection strategy avoids the bias of showcasing only the best individual cases, aiming to provide a more comprehensive and objective reflection of the model’s general performance. The figure illustrates the distribution of spatial attention weights when the model processes EEG signals from these five different participants.

As can be clearly observed in Figure 8, the model can adaptively focus on different brain regions for different subjects. For example, when processing data from certain subjects, attention weights are more concentrated in regions such as the prefrontal cortex or occipital lobe, while for other subjects, the focus of attention may differ. These differences in attention distribution across subjects strongly demonstrate that the CBAM does not use a fixed spatial filter but instead dynamically and adaptively identifies and enhances signals from key brain regions most closely associated with emotional states, while suppressing irrelevant or noisy regions, based on the unique characteristics of the input signals.

This visualization result provides a strong explanation for the model’s high performance. It is precisely through the effective spatial focusing of the CBAM that the model can accurately capture the most discriminative spatial features for emotion classification from the complex 62-channel EEG signal. This is highly consistent with the conclusion drawn from the ablation experiment, which showed a decline in performance after removing the CBAM.

### 3.4. Results on the DEAP Dataset

This section details the performance evaluation of the proposed TANet model on the DEAP dataset. Our analysis is centered on a comparative study against established benchmark models, complemented by a series of visualizations. The DEAP dataset, which forms the basis of this evaluation, consists of 32-channel EEG data from 32 participants. These signals were decomposed into five standard frequency bands (Delta, Theta, Alpha, Beta, and Gamma). To align our approach with the evaluation paradigms of similar advanced studies, as discussed in Section 3.2, we adopted a random segmentation strategy at the segment level. This process of partitioning the dataset at a 9-to-1 ratio was repeated for 10 independent runs using different random seeds to ensure a robust and reliable evaluation. The final results presented in this section are the mean and standard deviation derived from these 10 runs.

#### 3.4.1. Comparison with the Benchmark Model

To comprehensively validate the effectiveness and generalization ability of the TANet model on the DEAP dataset, we compared its performance with several benchmark models that have been used in EEG emotion recognition studies on the DEAP dataset: (1) DGCNN: a deep graph convolutional neural network that can effectively capture the inherent spatial topological relationships and functional connections between EEG electrodes, thereby extracting more discriminative spatiotemporal features [31]; (2) ACRNN: this convolutional recurrent neural network incorporates attention mechanisms. It is designed to adaptively focus on significant spatiotemporal information within EEG signals by using attention modules, while its recurrent layers are responsible for capturing temporal dependencies [37]; (3) CapsNet: a novel neural network architecture designed to capture hierarchical relationships between entities and their attributes through “capsule” units, used in EEG classification to better identify and express complex EEG patterns [38]; (4) 4D-CRNN: A four-dimensional convolutional recurrent neural network designed for EEG signals, capable of handling the multidimensional characteristics of EEG data, extracting local features through convolutional layers, and processing temporal dependencies through recurrent layers [39]; (5) EESCN: A network focused on EEG emotion recognition, which enhances the extraction and classification of emotion-related features through specific structures or mechanisms, aiming to improve the discriminative power of EEG signals [40]; (6) TSFFN: A spatiotemporal feature fusion network that integrates temporal and spatial features to enhance the performance of EEG emotion recognition [41]; (7) MCA&3D-CNN: Combines the mutual cross-attention mechanism with a 3D convolutional neural network. The MCA module aims to discover and fuse complementary relationships between features from different domains, such as differential entropy and power spectral density, while the 3D-CNN processes new three-dimensional feature representations of channel-frequency-time to achieve high-performance emotion recognition [22]. The selected benchmark models encompass a range of traditional machine learning methods and diverse deep learning architectures, chosen to thoroughly demonstrate the advantages of our proposed model. To comprehensively evaluate its classification capabilities, we utilized accuracy as the primary metric, calculated for both the valence and arousal dimensions. The results of these comparative experiments are summarized in Table 7.

The evaluation on the DEAP dataset was conducted under the segment-level evaluation paradigm to ensure a direct comparison with prior works, with results detailed in Table 7. Within this specific context, our proposed TANet model demonstrated highly competitive and stable performance. Across 10 independent runs, it attained a mean recognition accuracy of 99.69 ± 0.15% for the valence dimension and 99.67 ± 0.13% for the arousal dimension. This level of performance, coupled with the extremely low standard deviations, is further corroborated by comprehensive metrics, with both F1 scores and Kappa values also reaching approximately 0.99 for each dimension. When compared to benchmark methods under similar evaluation protocols, such as DGCNN, ACRNN, and EESCN, TANet shows a clear advantage, showcasing its robust architecture. We attribute this success to the powerful synergy of the model’s integrated triple-attention mechanism. Specifically, the Conformer module surpasses traditional models by effectively capturing complex temporal dynamics, while the CBAM and MCA modules enhance feature extraction by deeply refining and fusing information across spatial, channel, and cross-modal domains. This combination allows TANet to establish a strong performance level within this established comparative framework.

Similarly, to rigorously evaluate the contribution of each core component on the DEAP dataset, we performed statistical analyses comparing the full TANet model against each of its ablated versions. Paired *t*-tests on the accuracy scores from the 10 independent runs confirmed that the full model was significantly superior to all three ablated counterparts for both the valence and arousal dimensions, with a *p*-value below 0.001 for all comparisons. This provides robust statistical evidence that the Conformer, CBAM, and MCA modules each make an individually significant contribution, highlighting the synergistic effectiveness of our triple-attention architecture.

#### 3.4.2. Feature Distribution Visualization

**Figure 9:**
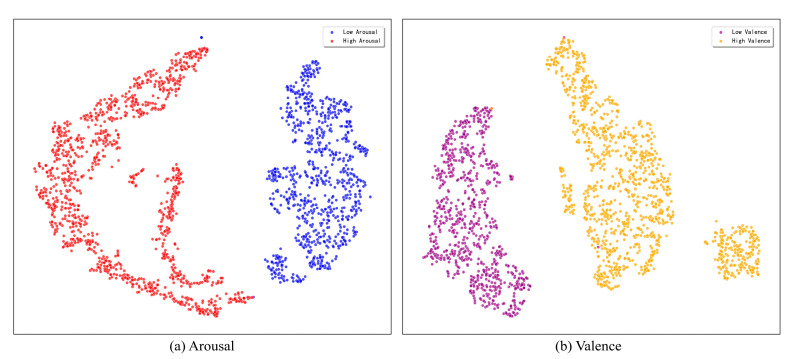
t-SNE dimensionality reduction visualization of features learned by a representative run of the TANet model on the DEAP dataset. (**a**) shows the arousal dimension, where red represents high arousal and blue represents low arousal; (**b**) shows the valence dimension, where yellow represents high valence and purple represents low valence.

This study utilizes the t-SNE dimensionality reduction technique to visually evaluate the discriminative power of the feature representations learned by the model. To illustrate the model’s discriminative power, we map the high-dimensional feature vectors from the classification layer of a representative single run onto a two-dimensional plane for this assessment. The resulting visualization, presented in Figure 9, indicates that the model exhibits exceptional feature learning capabilities for both the arousal and valence dimensions on the DEAP dataset.

As can be seen from the figure, on the arousal dimension, the sample points of high and low arousal categories form distinct clusters with clear boundaries and almost no overlap. Similarly, on the valence dimension, the sample points of high and low valence also exhibit high intra-class compactness and inter-class separability. This visualization result strongly demonstrates that the proposed triple attention network can learn highly separable emotional features from complex EEG signals, providing an intuitive explanation and support for its outstanding performance in classification tasks.

#### 3.4.3. Confusion Matrix Analysis

**Figure 10:**
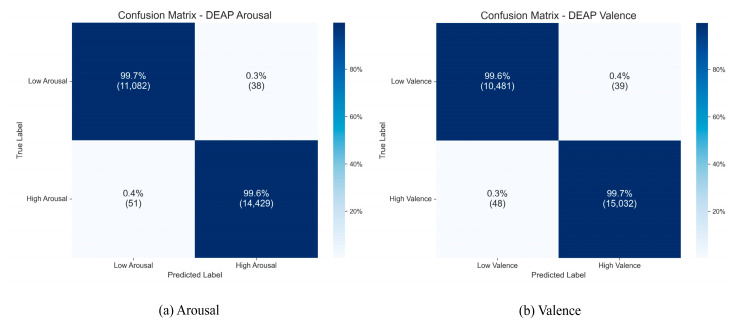
Aggregated and normalized confusion matrices of the TANet model on the DEAP dataset, compiled from 10 independent runs. (**a**) shows the arousal dimension; (**b**) shows the valence dimension. The diagonal values represent the prediction accuracy of each category, and the numbers in parentheses represent the total number of samples in that cell, summed across all 10 runs.

To gain a more precise understanding of the TANet model’s classification performance for each category on the DEAP dataset, we created aggregated confusion matrices from the 10 independent runs, which are displayed in Figure 10.

As the figure illustrates, the model attained consistently high recognition accuracies across all categories. For the Arousal dimension, the accuracies were 99.7% for the “low arousal” category and 99.6% for the “high arousal” category. On the Valence dimension, the model also achieved excellent recognition accuracy rates of 99.6% for the “low valence” and 99.7% for the “high valence” categories. The confusion matrices for both dimensions show that the diagonal elements, representing the cumulative results of 10 runs, are nearly perfect, while the misclassification rates for non-diagonal elements are extremely low. This clearly indicates that the model not only demonstrates superior overall performance but also exhibits extremely high classification accuracy and robustness across various subcategories of emotions.

#### 3.4.4. ROC Curve Analysis

To further quantify the model’s discriminative ability and stability on the DEAP dataset, we plotted the mean ROC curves and calculated the average AUC values from the 10 independent runs.

**Figure 11:**
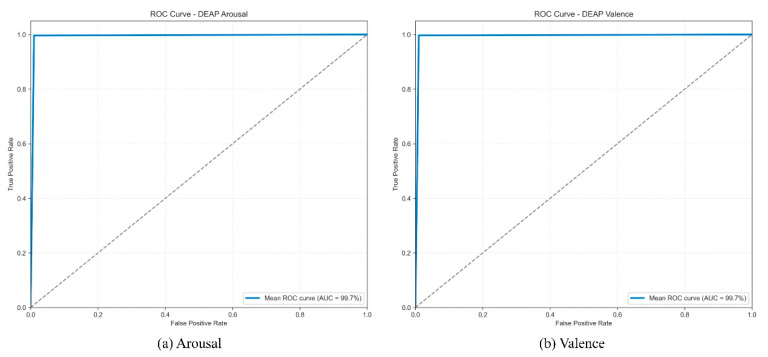
Mean ROC curves of the TANet model on the DEAP dataset, averaged from 10 independent runs. (**a**) shows the Arousal dimension and (**b**) shows the Valence dimension. Both dimensions achieved a mean AUC of 99.7%.

As shown in Figure 11, the mean ROC curves for both the Arousal and Valence dimensions closely follow the upper left corner, indicating consistently high performance. The model achieved a stable and extremely high mean AUC of 99.7% on both dimensions. These high average AUC scores demonstrate that the model possesses nearly perfect and reliable classification discriminative capability across different experimental runs. This conclusion is highly consistent with the analysis results of the t-SNE feature distribution and the aggregated confusion matrix, collectively validating the model’s robust capability to extract highly discriminative emotional features from complex EEG signals.

#### 3.4.5. Model Convergence Analysis

**Figure 12:**
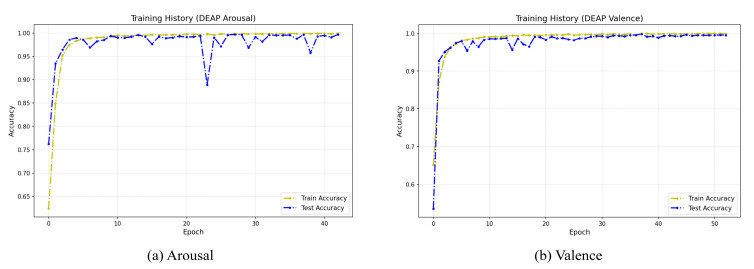
Training history curves from a representative single run of the TANet model on the DEAP dataset. (**a**) shows the arousal dimension, and (**b**) shows the valence dimension. The figure shows the changes in training accuracy and testing accuracy with the number of training iterations.

To evaluate the stability and convergence behavior of our model during the training phase, we visualized its training history on the DEAP dataset. Figure 12 illustrates the learning curves from a representative single run, which is indicative of the typical training pattern due to the high stability confirmed by our 10-run evaluation (Table 5). These curves clearly demonstrate that the model achieves rapid convergence on both dimensions. During the early stages of training (approximately the first 10 epochs), both training and test accuracy rapidly rise above 95% and quickly enter a high-performance stable plateau phase approaching 100%. Notably, throughout the training process, the test accuracy curve closely aligns with the training accuracy curve, with no significant gap between the two. This indicates that the model possesses excellent generalization capabilities and has not overfit on the training data, further validating the effectiveness of the model architecture and the rationality of the training strategy.

#### 3.4.6. Subject-Independent Analysis

The TANet achieves promising performance on subject specific emotion recognition problem. We further explore the possibility of extending it to subject-independent emotion recognition problem. Moreover, we take two state-of-the-art methods for comparison: MEET [42] and MASA-TCN [43]. Because the TANet is mainly designed for subject specific tasks, we use a one-subject-held-out setting in experiment. For example, the data of the first 31 subjects in the DEAP dataset is used for training; while the data of the last subject is used for testing. The experiment results are given in Table 8.

From Table 8, the TANet demonstrates competitive and stable performance compared to other approaches across valence and arousal tasks. On valence task, the TANet achieves the highest accuracy of 63.94 ± 3.83%, outperforming EEGNet, DeepConvNet, MEET, and MASA-TCN. On the other hand, TANet’s F1-score is lower than MEET and MASA-TCN. On arousal task, the TANet ties with MASA-TCN for the highest accuracy, surpassing EEGNet, DeepConvNet, and MEET. On the other hand, TANet’s F1-score is comparable to EEGNet and MASA-TCN, while having a much smaller standard deviation than all other methods.

## 4. Discussion

### 4.1. Principal Findings and Model Contributions

To advance the performance of emotion recognition from EEG signals, this paper introduces TANet, a new network architecture integrating a cross-modal attention module, a convolutional block attention module, and a Conformer module. The core advantage of this study lies in the effective synergistic interaction of the triple-attention mechanism, which was validated through ablation experiments. The results clearly reveal the indispensability of each module, with the Conformer playing a central role in capturing temporal dynamics, supported by MCA for cross-modal fusion and CBAM for spatial-channel refinement. It is this three-stage processing workflow that enables the model to learn highly discriminative feature representations.

Experimental analysis shows that the TANet model achieves strong and stable classification performance on both the SEED and DEAP public datasets. On the SEED dataset, the model achieves a mean accuracy of 98.51 ± 1.40% across subjects, significantly outperforming benchmark models. On the DEAP dataset, under a segment-level evaluation paradigm for fair comparison, the model also surpassed advanced methods with mean accuracy rates of 99.69 ± 0.15% (valence) and 99.67 ± 0.13% (arousal). These robust statistical results were intuitively validated through t-SNE visualization, which demonstrated the model’s strong feature discrimination capability.

### 4.2. Potential Applications

The TANet model’s potential for high-precision emotion recognition makes it highly promising for various practical applications. In the field of intelligent healthcare, it can serve as an objective auxiliary diagnostic tool for monitoring emotional fluctuations in patients with emotional disorders such as depression and anxiety. In the field of HCI, the model can be used to develop intelligent systems that can perceive user emotions and provide personalized feedback, such as adaptive learning software, intelligent driving assistance systems, and immersive virtual reality experiences.

### 4.3. Limitations and Future Work

To provide a comprehensive perspective on our findings, it is important to discuss the context and limitations of our evaluation protocols. The evaluation on the DEAP dataset, while ensuring a fair comparison with prior works, followed a segment-level protocol. We acknowledge that this setup primarily assesses within-trial pattern discrimination and is not designed for evaluating cross-subject generalization, a known challenge due to high inter-subject variability [44,45]. Future work must focus on validating TANet’s robustness under stricter subject-independent schemes, such as leave-one-subject-out (LOSO).

Furthermore, based on the discussion of the validation and information leakage issues [46,47], we recognize additional avenues for future improvement. While our evaluation on the SEED dataset employed a robust subject-specific cross-validation, exploring cross-session generalization would provide deeper insights into the model’s stability over time. From a practical standpoint, the computational complexity of the triple-attention architecture, while effective, warrants future investigation into model optimization and compression techniques to facilitate real-time deployment in resource-constrained applications. To promote reproducibility, detailed model configurations are provided in the Methods section, and our implementation code is available from the corresponding author upon reasonable request. These considerations are crucial for bridging the gap between strong theoretical performance and practical, generalizable BCI systems.

## 5. Conclusions

This paper proposes a novel network architecture named TANet, which consists of a cross-modal attention module, a convolutional block attention module, and a Conformer temporal modeling module. The model innovatively integrates the three attention mechanisms to comprehensively and deeply extract emotion-related features from EEG signals. Unlike traditional methods, this model can directly process pre-extracted DE and PSD features, achieving efficient emotion recognition through an end-to-end approach. Extensive experimental results on the SEED and DEAP public datasets demonstrate that the model achieves superior performance, significantly outperforming various existing benchmark methods. This study not only validates the effectiveness of the proposed model framework but also provides a promising new approach for developing high-performance, robust emotion-based brain–computer interface systems.

## Figures and Tables

**Table 1:** Description of the DEAP Dataset.

Project	Description
Dataset Name	DEAP
Number of Subjects	32 subjects
Experimental Design Data Type	Each subject watched 40 one-minute music videos 32-channel EEG signals
Task Type	Four dimensions: valence, arousal, dominance, and liking. This study focuses on a binary classification task for the valence and arousal dimensions
Data Shape Sampling Rate	(40,328,064) 128 Hz

**Table 2:** Description of the SEED Dataset.

Project	Description
Dataset Name	SEED
Number of Subjects	15 subjects
Emotion Categories	Positive, Negative, Neutral
Number of EEG Electrodes	62 electrodes
Sampling Rate	1000 Hz

**Table 3:** The TANet Model Architecture Details.

Module	Layer	Kernel Size	Output Shape
Input	DE Features	-	(B, F, C, T) → (B, 5, 62, 10)
	PSD Features	-	(B, F, C, T) → (B, 5, 62, 10)
MCA	Mutual Cross-Modal Attention	in_dim = 10, att_dim = 32	(B, 62, 10)
	Feature Stacking	dim = 1	(B, 5, 62, 10)
CBAM	CBAM	reduction = 4, k_size = 7	(B, 5, 62, 10)
CNN	Conv2d + BN + GELU	in = 5, out = 32, k = (3, 3)	(B, 32, 62, 10)
	Conv2d + BN + GELU	in = 32, out = 64, k = (3, 3)	(B, 64, 62, 10)
	AdoptiveAvgPool2d	output_size = (31, 10)	(B, 64, 31, 10)
Projection	permute & reshape	-	(B, 10, 1984)
	Linear + LayerNorm	in = 1984, out = 256	(B, 10, 256)
Conformer1	Feed Forward	-	(B, 10, 256)
	Multi-Head Self-Attention	heads = 8	(B, 10, 256)
	Conv1d	kernel_size = 5	(B, 10, 256)
	Feed Forward	-	(B, 10, 256)
Conformer2	Feed Forward	-	(B, 10, 256)
	Multi-Head Self-Attention	heads = 8	(B, 10, 256)
	Conv1d	kernel_size = 5	(B, 10, 256)
	Feed Forward	-	(B, 10, 256)
Classifier	Global Average Pooling	dim = 1	(B, 256)
	Linear + GELU + Dropout (*p* = 0.3)	in = 256, out = 128	(B, 128)
	Linear + GELU + Dropout (*p* = 0.2)	in = 128, out = 64	(B, 64)
	Linear	in = 64, out = 3	(B, 3)

**Table 4:** Ablation Study Results for Core Module Combinations on the SEED dataset.

Model Combination	Accuracy (%)	95% CI for Accuracy	F1-Score	Kappa	*p*-Value
Conformer + CBAM	97.35 ± 2.09	[96.29, 98.41]	0.973 ± 0.021	0.960 ± 0.032	<0.001
Conformer + MCA	97.38 ± 1.96	[96.39, 98.37]	0.973 ± 0.020	0.960 ± 0.030	<0.001
CBAM + MCA	97.22 ± 1.77	[96.33, 98.11]	0.972 ± 0.018	0.958 ± 0.027	<0.001
Conformer + CBAM + MCA	98.51 ± 1.40	[97.80, 99.22]	0.981 ± 0.015	0.972 ± 0.021	-

**Table 5:** Ablation Study Results for Core Module Combinations on the DEAP Dataset.

Model Combination	Emotional Dimension	Accuracy (%)	95% CI for Accuracy	F1-Score	Kappa	*p*-Value
Conformer+ CBAM	Arousal	95.95 ± 0.25	[95.80, 96.10]	0.961 ± 0.005	0.923 ± 0.005	<0.001
	Valence	94.99 ± 0.30	[94.80, 95.18]	0.951 ± 0.006	0.902 ± 0.012	<0.001
Conformer + MCA	Arousal	93.40 ± 0.45	[93.12, 93.68]	0.934 ± 0.009	0.868 ± 0.019	<0.001
	Valence	95.73 ± 0.28	[94.80, 95.18]	0.959 ± 0.005	0.918 ± 0.010	<0.001
CBAM + MCA	Arousal	84.74 ± 0.85	[84.21, 85.27]	0.853 ± 0.015	0.707 ± 0.025	<0.001
	Valence	84.10 ± 1.10	[83.42, 84.78]	0.848 ± 0.020	0.697 ± 0.040	<0.001
Conformer + CBAM + MCA	Arousal	99.67 ± 0.13	[99.59, 99.75]	0.997 ± 0.001	0.993 ± 0.003	-
	Valence	99.69 ± 0.15	[99.60, 99.78]	0.997 ± 0.002	0.993 ± 0.003	-

**Table 6:** Performance Comparison against State-of-the-Art Methods on the SEED Dataset.

Method	Accuracy (%)	Kappa	F1-Score
SVM	83.10	0.78	0.80
2D-CNN	89.50	0.85	0.87
DGCNN	91.50	0.86	0.88
EEGNet	93.02	0.89	0.91
RGNN	93.50	0.88	0.90
DeepConvLSTM	93.75	0.91	0.92
TSception	94.13	0.92	0.93
EEG Conformer	95.30	0.92	0.93
PGCN	96.93	0.91	0.92
TANet (Ours)	98.51 ± 1.40	0.981 ± 0.015	0.972 ± 0.021

Note: Performance for our proposed TANet model is reported as mean ± standard deviation across 15 subjects. Results for other benchmark methods are cited from their respective publications, where standard deviations were not provided.

**Table 7:** Performance Comparison against State-of-the-Art Methods on the DEAP Dataset.

Method	Valence (%)	Arousal (%)
DGCNN	92.55	93.50
ACRNN	93.72	93.38
CapsNet	93.89	95.04
4D-CRNN	94.22	94.58
EESCN	94.56	94.81
TSFFN	98.27	98.53
MCA&3D-CNN	99.49	99.30
TANet (Ours)	99.69 ± 0.15	99.67 ± 0.13

Note: Performance for our proposed TANet model is reported as mean ± standard deviation across 10 independent runs. Results for other benchmark methods are cited from their respective publications.

**Table 8:** Performance under subject-independent setting on the DEAP Dataset.

Method	Valence Accuracy (%)	Valence F1-Score	Arousal Accuracy (%)	Arousal F1-Score
EEGNet	58.45 ± 9.30	0.581 ± 0.136	59.85 ± 8.80	0.622 ± 0.171
DeepConvNet	59.30 ± 10.45	0.601 ± 0.136	59.53 ± 9.09	0.615 ± 0.172
MEET	59.44 ± 9.45	0.634 ± 0.122	60.37 ± 12.08	0.587 ± 0.244
MASA-TCN	60.20 ± 8.13	0.646 ± 0.126	62.09 ± 10.39	0.622 ± 0.193
TANet (Ours)	63.94 ± 3.83	0.577 ± 0.047	62.10 ± 4.63	0.618 ± 0.046

## Data Availability

The SEED dataset analyzed in this study is available from https://bcmi.sjtu.edu.cn/home/seed/seed.html (accessed on 8 May 2025) and the DEAP dataset is available from https://www.eecs.qmul.ac.uk/mmv/datasets/deap/ (accessed on 11 May 2025).

## Abbreviations

The following abbreviations are used in this manuscript:

BCI	Brain–Computer Interface
CBAM	Convolutional Block Attention Module
CNN	Convolutional Neural Network
DE	Differential Entropy
EEG	Electroencephalography
HCI	Human–Computer Interaction
LOSO	Leave One Subject Out
LSTM	Long Short-Term Memory
MCA	Mutual Cross-Modal Attention
PSD	Power Spectral Density
RF	Random Forests
RNN	Recurrent Neural Network
SVM	Support Vector Machine
TANet	Triple Attention Network
